# Effectiveness of Vestibular Rehabilitation in Improving Health Status and Balance in Patients with Fibromyalgia Syndrome: A Single-Blind Randomized Controlled Trial

**DOI:** 10.3390/biomedicines11051297

**Published:** 2023-04-27

**Authors:** Ana Belén Peinado-Rubia, María Catalina Osuna-Pérez, Irene Cortés-Pérez, Alicia Rojas-Navarrete, María del Rocío Ibancos-Losada, Rafael Lomas-Vega

**Affiliations:** 1 AFIXA Fibromyalgia Association, 23008 Jaén, Spain; abpr0003@red.ujaen.es; 2 Department of Health Sciences, University of Jaén, Campus Las Lagunillas s/n., 23071 Jaén, Spain; 3 FisioCenter-Úbeda Physiotherapy Clinic, 23400 Úbeda, Spain

**Keywords:** fibromyalgia, balance, health status, vestibular rehabilitation, exercise therapy

## Abstract

Background: Fibromyalgia Syndrome (FMS) is a highly prevalent health problem whose main symptom is widespread pain, although it presents as other manifestations, such as loss of balance, that seem to mainly affect visuo-vestibular information. Objective: to compare the effects of a Vestibular Rehabilitation (VR) program versus those of a Conventional Physical Exercise (CPE) program on the health status of patients with FMS. Methods: A single-blind randomized controlled trial was performed. Patients with FMS were randomly assigned to VR or CPE programs. The protocols were performed in 40 min group sessions, twice weekly, for 16 sessions. Perceived health status, static and dynamic balance, verticality perception, confidence in balance, sensitization and kinesiophobia were measured at baseline, post-treatment and at the three-month follow-up and analyzed using an intention-to-treat approach. Results: Forty-eight subjects were randomly assigned, of whom thirty-five completed the planned VR (n = 19) or CPE (n = 16) program. At the three-month follow-up, there were differences in physical health status measured with the SF-12 (mean = −4.36, SE = 1.88, *p* = 0.027), balance during walking (mean = 1.90, SE = 0.57, *p* = 0.002), the perception of verticality in degrees (mean = 3.61, SE = 1.51, *p* = 0.024) and the anteroposterior position of the center of pressure (mean = −7.88, SE = 2.80, *p* = 0.009), as well as a decrease in the number of falls (mean = 0.98, SE = 0.44, *p* = 0.033), favoring the VR group. Conclusions: Vestibular Rehabilitation can be as beneficial as conventional exercise in improving the state of health in patients with Fibromyalgia Syndrome, providing additional improvements in physical health status, body balance, the perception of verticality and the number of falls.

## 1. Introduction

Fibromyalgia is considered a central pain syndrome characterized by a sensory processing disorder [1]. Those affected by Fibromyalgia Syndrome (FMS) mainly have a general hypersensitivity to painful stimuli triggered by adaptive changes in the central nervous system, a process known as Central Sensitization (CS) [2,3]. The sensitization process could extend to other sensory systems, such as the vestibular, visual or somatosensory system, producing symptoms of dizziness and instability [2] or a deficit in postural control [4], which are common comorbidities in FMS. 

Balance disturbance represents one of the ten most debilitating symptoms for this population, with prevalence between 45% [5] and 68% [6]. It has been described that the use of maladaptive sensory strategies can produce postural instability, blurred vision before head movements, dizziness or imbalance [7]. FMS patients have deteriorated postural control, which worsens in situations of sensory input disturbances, and a higher prevalence of falls compared with healthy controls [8,9]. Other recent studies have shown a specific impairment in the central integration of visuo-vestibular information in patients with fibromyalgia [10], as well as a greater inability to maintain balance during head movements [11]. In addition, it has previously been suggested that the perception of dizziness in these patients seems to be associated with a greater impact of the disease and a substantial worsening of the perception of quality of life [12]. Dizziness symptoms can cause anxiety and fear, leading to avoidance of activities and reduced mobility [13]. The impact of symptoms such as dizziness and postural instability on FMS can be considerable due to the chronic and persistent nature of the condition, as well as its negative consequences on the activities of daily living and general health status of these patients [8,9]. The main complaints of global health status in Fibromyalgia Syndrome (physical function, work difficulties, depression, anxiety, sleep, pain, stiffness, fatigue and well-being) should be collected and presented in the main instruments to assess the impact of this disease [14,15,16].

The available literature supports that physical exercise improves physical function and balance in patients with FMS [17,18]. Many of these conventional physical exercise programs are fundamentally based on static postures and dynamic movements that combine stretching and muscle activation [18]. However, these exercises cannot be categorized as Vestibular Rehabilitation therapy. Antidepressant medication prescriptions such as Selective Serotonin Reuptake Inhibitors (SSRIs) have also been used as pharmacological treatments for the improvement of chronic dizziness [19]. Vestibular Rehabilitation (VR) is used in functional vestibular disorders [20] and pretends to promote vestibular compensation by focusing on exercises that improve postural stability and facilitate habituation to conflicting sensory inputs (own movements or environmental situations that cause symptoms) in order to desensitize the patient, increase their tolerance to these sensory stimuli [21], decrease restrictions on activities of daily living and encourage social participation [13]. In addition, several studies have also reported improvements in subjective dizziness and postural control after VR supervised and delivered by physiotherapists [20,22].

FMS patients could benefit from a Vestibular Rehabilitation (VR) program based on movement desensitization exercises. VR is an effective and safe treatment that improves balance, functional recovery, emotional states and quality of life and decreases the risk of falls and dizziness [7,23,24]. Current knowledge corroborates the use of VR in functional vestibular disorders of central origins [7,24,25], being also a cost-effective treatment [23]. Based on these findings, the purpose of this study was to analyze the effectiveness of a VR program to improve balance and health status in patients with FMS, as well as to determine the therapeutic effects after VR therapy in the medium term.

## 2. Materials and Methods

### 2.1. Design

A multicenter controlled single-blind randomized clinical trial with two parallel groups was conducted. The study was approved by the Ethics Committee of the University of Jaén (JUN.21/8.TES). The purpose and the procedure of the study were conducted under the guidelines of the Declaration of Helsinki. 

Trial registration: ClinicalTrials.gov (identification number: NCT05300529)

### 2.2. Participants

The sample was recruited through the Fibromyalgia Association of the city of Jaén (AFIXA). Potential participants were contacted by phone and email to provide detailed information about the characteristics of the study and their participation in it. From April to September 2021, potential participants were assessed for eligibility. Fibromyalgia Association patients from different municipalities in the province of Jaén agreed to participate in the study. The inclusion criteria were: (a) aged between 18 and 70 years and (b) a diagnosis of Fibromyalgia Syndrome according to the 2016 American College of Rheumatology (ACR) criteria. Those who presented any of the following conditions were excluded: (a) a cognitive deficit, (b) an inability to stand autonomously, (c) a severe visual deficit, (d) vestibular disease, (e) neurological disease with balance impairment or (f) orthopedic disorders with impaired standing and locomotion. Written informed consent was obtained from all participants.

### 2.3. Sample Size Calculation

Because this study is the first to analyze the effectiveness of VR in patients with FMS and in accordance with current recommendations for sample size in pilot studies, to achieve a power of 80% with an alpha error of 5%, under the precision and efficiency considerations, a total of 20 subjects per group would be required [26,27]. Taking into account a dropout ratio of 16%, 48 subjects were finally selected to participate in the study.

### 2.4. Randomization

Stratified random assignment was performed to generate balanced groups using the Epidat 3.1 program. The blocks were made according to the treatment center closest to the patients’ places of residence (Jaén capital or Torredelcampo). The investigator responsible for this procedure was blinded to the group assignments during the randomization and intervention processes and did not participate in the recruitment or evaluation of the participants. Only the physiotherapists providing the intervention knew the allocation of the groups and the distribution of the subjects. Each participant belonged to an intervention group without being able to identify or differentiate which was the target intervention of this study. Statistical analysis was performed by a statistical researcher who was not involved in the procedure.

### 2.5. Interventions

The experimental intervention was carried out simultaneously in two locations in the province of Jaén (Jaén capital and Torredelcampo). The protocols were implemented by physiotherapists with ten years of experience in the management of patients with FMS and balance disorders. All participants received the same treatment dosage: 2 clinical sessions per week led by a physiotherapist for 8 weeks (16 sessions in total), with each session lasting 40 min. As a complementary treatment, the participants performed home exercises according to their action protocol, 2 times a day, 5 to 7 days per week [28,29]. Participants were informed about all stages of treatment, the importance of daily exercise and the possibility of increased dizziness, especially in the early stages of rehabilitation. It was recommended that the participants maintain their usual activities [29] and go for a daily walk [30]. The patients were asked to attend at least 50% of the therapeutic sessions (8/16).

### 2.6. Intervention Protocols

Participants in the intervention group received VR based on movement desensitization models. The VR intervention consisted of Gaze Stabilization Exercises (GSEs) based on adaptation and substitution concepts, exercises for balance training associated with eye–head movements, exercises for improving postural stability in challenging sensory conditions and exercises for dynamic balance training focused on walking exercises with head movements. Two levels of difficulty were established in the VR intervention. The progression of the exercises was determined by variations in posture, speed, exposure time and patient tolerance. The VR protocol was developed from the clinical experiences of researchers and recommendations from the scientific literature [7,24,28,31]. The Vestibular Rehabilitation program can be downloaded from Supplementary Material. Participants in the control group received Conventional Physical Exercise (CPE) therapy based on physical maintenance exercises, joint mobility exercises and flexibility exercises.

### 2.7. Measurements

Measurements were performed at three time points: a pre-randomization assessment at the initiation of the intervention (T0), an assessment at the end of the intervention (T1) and an assessment at the three-month follow-up (T2). Sociodemographic data were collected at baseline. Clinical data were extracted from medical records at T0, T1 and T2. Primary outcome measures were health status assessed with the Fibromyalgia Impact Questionnaire and balance assessed with the Dizziness Handicap Inventory and the Activities-specific Balance Confidence Scale. All study variables classified into self-reported outcome measures or objective outcomes are presented below.

#### 2.7.1. Self-Reported Outcome Measures

The Spanish version of the Fibromyalgia Impact Questionnaire (FIQ) [32] is used to measure the impact of a disease. The FIQ comprises 10 items that measure physical disability and the degree of specific symptoms, such as pain, stiffness, fatigue, depression and anxiety and general well-being during the past week. Each symptom is measured on a response scale from 0 (no symptoms) to 10 (very severe), with total scores ranging from 0 to 100. The Spanish version of the FIQ shows a high internal consistency (α = 0.80).

The Numerical Pain Rating Scale (NPRS) [33] is used to measure the intensity of pain described by a patient using a horizontal line of 10 cm, where 0 indicates the absence of pain and 10 the most intense pain perceived.

The Spanish version of the Short Form Health Survey (SF-12) [34] is used to analyze the physical and mental health of participants by two summary scores: the Physical Component Summary (PCS) and Mental Component Summary (MCS). The SF-12 is a shortened version of the SF-36 and has an algorithm that allows health scoring in these two dimensions on a scale from 0 to 100.

The Spanish adaptation of the Central Sensitization Inventory (CSI) [35] is used to quantify the level of central sensitization, with 25 items with a wide range of somatic and emotional symptoms. It has a total range of 0 to 100, where higher scores indicate a greater level of sensitization. The Spanish version of the CSI shows a high internal consistency (α = 0.872) and a one-dimensional factorial structure.

The Fatigue Severity Scale (FSS) in its Spanish version [36] is used to evaluate the impact of fatigue, with 9 items that assess aspects of physical and mental fatigue and social aspects. It ranges from 9 to 63, where scores ≥ 36 indicate severe fatigue.

The Spanish adaptation of the Pain Catastrophizing Scale (PCS) [37] is used to measure the level of catastrophism, with 13 items that evaluate catastrophic thoughts with a response scale from 0 (none/never) to 4 (all the time) and a total score ranging from 0 to 52 points. Higher scores indicate a greater presence of catastrophic thoughts. The Spanish version of the PCS shows excellent internal consistency (α = 0.92).

The Spanish version of the Tampa Scale of Kinesiophobia (TSK) [38] is used to determine the fear of movement, with 11 items that evaluate the fear of movement or injury with a response scale from 1 (strongly disagree) to 4 (totally agree) and a total score ranging from 11 to 44 points, where high scores indicate increased fear of movement. The Spanish version of the TSK shows good internal consistency (α = 0.78).

The Spanish version of the Dizziness Handicap Inventory (DHI) [39] is used to quantify perceived disability in patients with vertigo, dizziness or instability and its impact on activities of daily living. It consists of 25 items divided into three separate subscales (the physical (DHI-P), emotional (DHI-E) and functional (DHI-F) subscales), with total scores ranging from 0 to 100 points, where higher scores indicate a greater degree of disability. The Spanish version of the DHI shows high internal consistency (α = 0.87).

The Spanish adaptation of the Activities-specific Balance Confidence Scale (ABC-16) [40] is used to assess the level of confidence when performing a specific task without losing balance, with 16 questions with a scale of ascending answers from not at all confident (0%) to completely safe (100%) and a total score ranging between 0 and 100%. Values below 67% have proven to be sensitive and specific for the prediction of falls, as well as for the considerable reduction of independence. The Spanish version of ABC-16 shows excellent internal consistency (α = 0.916). 

The Spanish version of the Falls Efficacy Scale International (FES-I) [41] is used to assess fear of falling, with 16 items that record the level of concern about falls during activities and inside and outside the home, with a response scale ranging from 1 (not at all worried) to 4 (very worried) and a total score ranging from 16 to 64 points, where high scores are associated with a greater fear of falling. The Spanish version of the FES-I shows excellent internal consistency (α = 0.940).

The number of falls suffered in the last 3 months was recorded by asking participants to answer the question “How many falls have you suffered in the last 3 months?”; a fall is described as an “unexpected event that causes the person to fall to the ground against his or her will” [42].

#### 2.7.2. Objective Measures

The Joint Assessment of Equilibrium and Neuro-motor Scale (JAEN Scale) [11] is used to assess the balance of patients with FMS. It consists of 20 functional balance tests with response alternatives classified into five categories ranging from no balance problem (0 points) to a complete or total balance problem (4 points) and a total score of 0 to 80 points, where a higher score indicates a higher degree of balance disorder. In addition to the total score, four subscales that measure instability during Head Movement (HM), When Support Is Reduced (SR), during Gait with Eyes Open (GEO) and Standing and Walking with Eyes Closed (SWEC) can be used. The perception of Visual Verticality (VV) is evaluated through the Subjective Visual Vertical Test (SVV) [43] and the Rod and Frame Test (RFT) [44]. The procedure used has been previously described [45]. In the RFT, the contribution of the visual system to the construction of the sense of verticality is analyzed [35]. The patient must vertically adjust a luminous line that projects inside an inclined frame with respect to the gravitational vertical on a dark background, estimating a correct perception of the vertical visual analyzed with the RFT, with values ranging from ±4.5° [46].

To measure static balance, a platform with a resistive pressure sensor with a surface of 400 × 400 mm and an acquisition frequency of 40 Hz was used and FreeStep© Standard 3.0 software (Sensor Medica, Rome, Italy) was used. The procedure is described in Romero-Franco et al., 2013 [47]. The posturographic parameters were: the area covered by the displacement of the Center of Pressure (CoP) (area, mm^2^); the CoP movement speed (V, mm/s); the CoP dispersion parameters in the mediolateral (RMSX) and anteroposterior (RMSY) (mm) directions; two CoP position parameters, including the mean CoP on the X axis and the mean CoP on the Y axis.

### 2.8. Statistical Analysis

Data are described by the mean and standard deviation for continuous variables and by frequencies and percentages for categorical variables. To measure baseline sociodemographic differences between the groups, the chi-square test for categorical variables and Student’s *t* test for continuous variables were used. To measure between-group differences at the end of treatment and at the 3-month follow-up, we used analysis of covariance with baseline scores as covariables. Effect size was measured with eta square, which is the equivalent of the coefficient of determination (R^2^) for experimental studies and could be interpreted as the proportion of between-group differences due to treatment. According to Cohen [48], an eta squared <0.02 can be considered insignificant, small if it is between 0.02 and 0.15, medium if it is between 0.15 and 0.35 and large if it is >0.35. The Statistical Package for Social Sciences (SPSS version 21) was used, with a confidence level of 95% (alpha error of 5%).

Per-Protocol (PP) analysis was performed using the scores of patients who completed treatment and had measurements; Intention-To-Treat (ITT) analysis was performed by estimating scores for those who did not complete treatment. Estimation Maximization (MS) was used to simulate missing values.

## 3. Results

**Figure 1:**
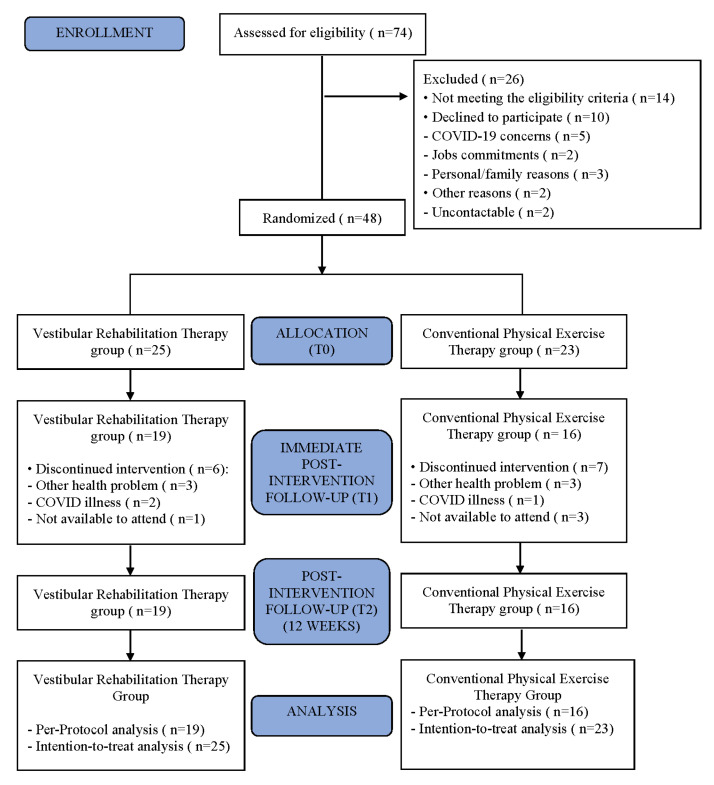
Flowchart.

A total of 48 patients were included in the study and randomly assigned to treatment groups (CPE: n = 23; VR: n = 25), all of whom completed baseline measurements (T0). Ultimately, 35 patients (n = 16 CPE; n = 19 VR) completed the treatment protocol (≥8/16 sessions performed) and the planned posttreatment evaluations (T1 and T2). A total of 13 subjects (27%) were lost to follow-up. A flowchart of patient selection and participation is shown in Figure 1.

Table 1 shows the comparability of the subjects’ sociodemographic variables. It was observed that the groups were comparable, except for slight differences in the limit of statistical significance in age. Table 2 shows the baseline comparability of the groups in the main outcomes. The groups were similar, except for slight differences in the PCS of the SF-12 and the RMSY with eyes open test.

Table 3 shows the between-group differences at the end of treatment with the significance obtained by the analysis of covariance. It was observed that in general, there was an improvement in both groups; however, the VR group had greater improvement in the global score of the JAEN scale, as well as for the subscales of Head Movements (JAEN-HMs) and Gait with Eyes Open (JAEN-GEO). The effect size could be considered medium for the JAEN total score and JAEN-Head Movement subscale and large for the JAEN-Gait with Eyes Open subscale.

Table 4 shows the between-group differences at the three-month follow-up. It was observed that in general, there was an improvement in both groups; however, there was greater improvement in the VR group for the PCS of SF-12, reduction of falls and JAEN gait subscale, a decrease in the degrees of deviation measured with the RFT and a greater anterior–posterior displacement of the Centre of Pressure with eyes closed. All effect sizes were considered medium.

In the ITT analysis, Little’s MCAR test showed a χ2 e value of 15,434,876,760 (*p* < 0.001); therefore, the values could not be considered Completely Missing At Random (MCAR) and Expectation Maximization (EM) was the technique selected to simulate the missing values.

In this analysis, at the end of the treatment, differences favoring the VR group were found in the JAEN total score (*p* = 0.029), the JAEN-Head Movement subscale (*p* = 0.015), the JAEN-Gait with Eyes Open subscale (*p* = 0.001) and, in the limits of significance, for the FIQ (*p* = 0.054). At the 3-month follow-up, significant differences favoring the VR group were found in Falls (*p* = 0.033), the JAEN- Gait with Eyes Open subscale (*p* = 0.036), the mean CoP Y axis with Eyes Closed subscale (*p* = 0.048) and, in the limits of significance, on the PCS of the SF-12 (*p* = 0.067), JAEN total score (0.056), JAEN-Standing and Walking with Eyes Closed subscale (*p* = 0.067) and RFT (0.080). However, the CSI (*p* = 0.032), and at the limits of significance for the V (0.056), RMSX (*p* = 0.056) and RMSY (*p* = 0.056), all with open eyes, favored the CPE group.

## 4. Discussion

In this study, we aimed to analyze the effectiveness of a VR program on improving the health status and balance of patients with FMS compared with a CPE program. The main finding of our study was that VR provides a greater improvement in balance during gait at the end of treatment. Additionally, the difference in the evolution of the outcome variables was consolidated at three months, encompassing both the state of physical health, the static balance measured with posturography, the dynamic balance measured with the JAEN scale, the perception of visual verticality and especially with a decrease in the frequency of falls. Furthermore, these results were confirmed overall after an intention-to-treat analysis. To our knowledge, this is the first study to explore the effects of VR on fibromyalgia patients. For ethical reasons, no pure control group was used against which to compare the actual effect of VR.

VR is a recommended therapy for the treatment of dizziness and imbalance in patients with central and functional vestibular disorders [49]. Current evidence identifies FMS as a multisensory syndrome [50]. Symptoms of vertigo and dizziness, described as instability, are reported by patients with FMS [8,51,52]; however, the clinical characterization of dizziness in FMS patients is still poor. A recent study [50] yielded very illuminating data on the prevalence of vestibular symptoms in this population (dizziness, 63.6%; migraine, 65.8%; visual fog, 80%). Migraine has been shown to affect disorders related to vestibular aspects (e.g., vestibular migraine or chronic dizziness) [53]. This statement further supports the need for data on the vestibular component in the pathophysiology of FMS. Dizziness represents a significant risk of falls, entailing large direct and indirect costs [7]. According to Sarihan et al. [54], the average rate of falls in FMS patients is 45%. In our study, we found improvements in the variables measured in both treatment protocols (VR vs. CPE); however, the VR group showed additional benefits in different outcomes, such as the improvement of dynamic balance measured with the total score of the JAEN scale, as well as those of its subscales, including the improvement of balance with cephalic movements and balance during walking, as well as static balance in the anteroposterior axis of the CoP, measured with static posturography. However, the most interesting clinical result of our study may be the decrease in the number of falls after 3 months of follow-up. FMS has been widely associated with an increased risk of falls [23,54,55], as well as the fear of suffering falls [55], due to the balance deficit present in these patients. Our findings on fall reduction coincide with those found by Rossi-Izquierdo et al. [56], who found a significant reduction in falls in the VR group versus the practice of daily physical activity (going for a walk) in older adult patients. However, thus far, few studies have tried to identify tools to improve this outcome, with no positive effects reported to date. Therefore, this is the first study to identify a therapy capable of reducing the number of falls in patients with FMS.

The perception of visual verticality improved with both therapeutic programs; however, only significant differences were found in the RFT in favor of the VR group. The RFT is used to analyze the contribution of the visual system to the construction of the sense of verticality (evaluation of visual dependence) [57]. High levels of visual dependence have been found in several groups of patients [58,59,60]. High visual dependence increases the risk of dizziness, lightheadedness, instability and disorientation in visually stressful environments [61]. This is the first time that this measure has been analyzed in FMS patients. The results of our study are in line with the literature, which establishes that greater visual dependence is a response to vestibular or proprioceptive deficiencies [62]; some authors have previously suggested that cervical proprioception could be related to other clinical variables of this syndrome and should be measured in subjects with FMS [63,64]. Despite the beneficial effects of VR for improving balance observed in our study, other physical exercise programs, such as those carried out in our control group, have shown effectiveness in improving health status [65,66] and balance [67] in patients with FMS. In our study, the change scores of the different variables were somewhat better in the VR group, although statistically significant results were not achieved. However, for the presence of central sensitization, better field scores were found in the conventional exercise group. In favor of the findings for the CPE group, it has been reported that the fear of dizziness can trigger behaviors of avoidance to movement and limitation of activities, thus increasing the general sensitization of the subject to any stimulus of the environment or self-generated. This situation, in turn, delays vestibular compensation and reduces long-term quality of life [68]. Some studies have suggested that, regardless of the VR protocol, the promotion of physical exercise in patients with chronic dizziness accelerates their functional recovery and improves quality of life [29,69]. This approach could justify decreasing central sensitization among our control group participants. Regarding physical health, we observed an improvement in the Physical Component Summary (PCS) of the SF-12 for the VR group compared with the CFE group. However, this effect was not detected in the Mental Component Summary (MCS). Previous studies have recommended combining vestibular rehabilitation with other therapeutic strategies, such as education in pain neuroscience or vertigo [70,71,72], relaxation techniques [72] or acupuncture [73], to more successfully address psychological aspects such as anxiety, kinesophobia or catastrophism, and the realization of physical exercise to contribute to the improvement of vertiginous symptomatology and provide well-being [18,74]. For future lines of work, it would be interesting to include a combined care of VR, therapeutic education and physical exercise to seek a complementarity of the different therapies and cover the wide range of symptoms that characterize FMS.

This study has several limitations. First, its pioneering nature limited the accuracy of sample size calculation. This, together with the loss of the subjects due to the natural morbidity of the disease and the restrictions due to the COVID-19 pandemic, resulted in a small final sample. However, the study is tremendously novel and can serve as a pilot for future studies to analyze the effects of this therapeutic modality in FMS.

## 5. Conclusions

Based on the results of this study, it can be concluded that Vestibular Rehabilitation is a therapeutic modality that can be used in patients with Fibromyalgia Syndrome to improve physical health, dynamic balance and verticality perception. The improvement in balance that is verified with the use of Vestibular Rehabilitation also reduces falls during the three months prior to the evaluation, being the first therapeutic modality that is shown to be effective for this purpose. Vestibular Rehabilitation, however, is no better than a conventional exercise program for improving mental health, disease impact or catastrophizing processes, kinesiophobia, central sensitization or balance confidence. Since these other variables can be addressed with other therapeutic alternatives, new studies should analyze the effects of including Vestibular Rehabilitation within multimodal therapeutic programs to search for a broader therapeutic effect.

## Figures and Tables

**Table 1:** Sociodemographic characteristics of the groups.

		CPE Group (n = 23)		VR Group (n = 25)		*p*-Value
		F	%	F	%	X^2^ tests
Gender	Female	22.0	95.7	24.0	96.0	0.734
	Male	1.0	4.3	1.0	4.0	
Civil Status	Single	0.0	0.0	2.0	8.0	0.107
	Married	20.0	87.0	14.0	56.0	
	Divorced	2.0	8.7	7.0	28.0	
	Widow	1.0	4.3	2.0	8.0	
Education	No studies	2.0	8.7	0.0	0.0	0.225
	Primary	10.0	43.5	7.0	28.0	
	Secondary	7.0	30.4	13.0	52.0	
	University	4.0	17.4	5.0	20.0	
Occupation	Active	5.0	21.7	9.0	36.0	0.551
	Sick leave	3.0	13.0	5.0	20.0	
	Unemployed	4.0	17.4	3.0	12.0	
	Housewife	1.0	4.3	2.0	8.0	
	Retired	10.0	43.5	6.0	24.0	
Dizziness last 3 months	Never	0.0	0.0	1.0	4.0	0.430
	Ever	9.0	39.1	14.0	56.0	
	Frequently	9.0	39.1	7.0	28.0	
	Always	5.0	21.7	3.0	12.0	
		Mean	SD	Mean	SD	*t* Test
Age		55.48	6.85	51.24	6.06	0.028 *
Weight		78.22	18.62	76.32	15.94	0.706
Height		159.17	6.53	162.84	5.92	0.047
Body Mass Index		30.88	7.09	28.86	6.28	0.299

CPE—Conventional Physical Exercise; VR—Vestibular Rehabilitation; F—Frequencies; %—Percentages; X^2^ tests—Chi Square test; SD—Standard Deviation. * *p* < 0.05.

**Table 2:** Baseline comparability.

	CPE Group (n = 23)		VR Group (n = 25)		*t* Test
	Mean	SD	Mean	SD	*p*-Value
Falls Last Three Months	1.13	1.18	1.40	1.47	0.490
FIQ TOTAL	66.02	15.78	67.50	12.89	0.723
NPRS	6.17	2.23	5.84	1.72	0.563
PCS of SF-12	39.09	4.46	42.74	4.20	0.005 **
MCS of SF-12	24.48	8.99	24.42	7.36	0.981
FSS	52.61	8.43	52.60	7.48	0.997
CSI TOTAL	64.39	14.72	63.72	7.86	0.843
TSK	29.22	7.43	26.52	6.36	0.182
PCS	28.74	15.16	24.92	10.95	0.319
DHI TOTAL	62.04	26.48	52.92	18.00	0.166
ABC-16	52.53	36.69	51.78	17.41	0.927
FES-I	39.48	12.27	34.64	9.20	0.127
JAEN Scale	40.00	11.92	34.60	10.09	0.096
SVV	4.20	6.39	2.55	1.41	0.214
RFT	11.34	9.12	11.84	11.45	0.869
Sway Area EO	513.46	651.96	309.88	511.23	0.233
Velocity EO	21.29	5.02	18.59	5.07	0.070
RMSX EO	0.39	0.10	0.36	0.10	0.312
RMSY EO	0.46	0.12	0.38	0.12	0.018 *
Mean CoP X axis EO	−2.51	6.71	−0.64	6.39	0.328
Mean CoP Y axis EO	−20.30	10.97	−15.87	10.76	0.165
Sway Area EC	692.49	971.60	607.83	743.55	0.737
Velocity EC	24.66	6.04	21.52	5.50	0.069
RMSX EC	0.48	0.18	0.42	0.14	0.200
RMSY EC	0.57	0.18	0.49	0.15	0.081
Mean CoP X axis EC	−2.58	7.85	−0.15	4.93	0.205
Mean CoP Y axis EC	−17.42	13.98	−15.74	12.18	0.661

CPE—Conventional Physical Exercise; VR—Vestibular Rehabilitation; SD—Standard Deviation; FIQ—Fibromyalgia Impact Questionnaire; NPRS—Numerical Pain Rating Scale; PCS of SF-12—Physical Component Summary of The 12-Item Short Form Health Survey; MCS of SF-12—Mental Component Summary of The 12-Item Short Form Health Survey; FSS—Fatigue Severity Scale; CSI—Central Sensitization Inventory; TSK—Tampa Scale of Kinesiophobia; PCS—Pain Catastrophizing Scale; DHI—Dizziness Handicap Inventory; UCLA-DQ; ABC-16—Activities-specific Balance Confidence Questionnaire; FES-I—Falls Efficacy Scale International; JAEN Scale—Joint Assessment of Equilibrium and Neuro-motor Scale; SVV—Subjective Visual Vertical Test; RFT—Rod and Frame Test; EO—Eyes Open; RMSX—Root Mean Squared calculated by X axis position values; RMSY—Root Mean Squared calculated by Y axis position values; CoP—Center of Pressure; EC—Eyes Closed; * *p* < 0.05; ** *p* < 0.01.

**Table 3:** Between-group differences by analysis of covariance using baseline scores as covariate. Per-Protocol analysis at the end of treatment.

	CPE Group (n = 16)		VR Group (n = 19)		Differences		Effect Size	
	Mean	SD	Mean	SD	Mean	SE	*p*-Value	ETA^2^
FIQ	67.64	14.39	62.15	13.52	6.94	4.18	0.108	0.090
NPRS	5.81	1.38	5.63	1.95	0.27	0.42	0.513	0.013
PCS of SF-12	41.10	6.75	43.25	5.80	−0.50	2.20	0.824	0.002
MCS of SF-12	27.38	10.59	25.46	8.35	0.94	2.86	0.745	0.003
FSS	45.50	12.05	47.32	8.13	−1.62	2.70	0.553	0.011
CSI	58.88	12.09	59.32	7.29	1.98	2.52	0.439	0.019
TSK	28.00	7.56	26.58	4.83	1.35	1.54	0.386	0.024
PCS	24.38	12.75	24.16	13.04	0.05	2.84	0.986	0.000
Falls	1.13	0.27	0.52	0.25	0.60	0.38	0.118	0.075
DHI-Total	52.88	28.71	47.26	19.37	2.78	4.89	0.573	0.010
DHI Emotional	16.38	11.18	12.42	7.82	1.05	1.69	0.540	0.012
DHI Functional	19.38	10.78	17.37	7.03	1.63	1.98	0.416	0.021
DHI Physical	17.13	8.03	17.47	6.10	0.20	1.97	0.918	0.000
ABC-16	55.59	20.26	58.45	18.60	−6.94	5.69	0.232	0.044
FES-I	34.13	9.98	31.05	7.32	0.99	2.06	0.633	0.007
JAEN-Total Score	34.75	12.95	22.84	10.31	7.74	3.45	0.032 *	0.136
JAEN-HM	6.88	4.54	3.42	3.24	3.17	0.97	0.002 **	0.252
JAEN-SR	21.00	6.50	15.84	6.03	2.51	2.12	0.246	0.042
JAEN-GEO	4.13	2.87	1.16	1.30	2.90	0.66	0.000 ***	0.379
JAEN-SWEC	2.75	1.91	2.42	1.30	0.18	0.56	0.748	0.003
SVV	2.55	1.52	2.40	1.26	0.07	0.52	0.892	0.001
RFT	12.48	13.75	8.52	8.82	3.25	2.71	0.241	0.051
Sway Area EO	261.67	294.08	194.01	281.22	−48.51	89.48	0.592	0.010
Velocity EO	17.58	4.70	16.54	4.37	0.09	1.43	0.951	0.000
RMSX EO	0.36	0.08	0.32	0.07	0.03	0.03	0.189	0.059
RMSY EO	0.35	0.12	0.33	0.12	−0.02	0.04	0.550	0.012
Mean CoP X axis EO	−3.73	7.37	−0.62	6.32	−3.20	2.22	0.161	0.067
Mean CoP Y axis EO	−19.71	8.20	−14.93	6.79	−4.09	2.82	0.158	0.068
Sway Area EC	815.84	1170.98	508.33	1078.73	92.49	375.95	0.807	0.002
Velocity EC	19.90	5.68	19.43	6.70	−1.03	1.92	0.593	0.010
RMSX EC	0.40	0.13	0.38	0.13	−0.01	0.04	0.866	0.001
RMSY EC	0.46	0.12	0.45	0.21	−0.02	0.06	0.721	0.004
Mean CoP X axis EC	−6.87	14.79	−1.00	5.28	−5.21	3.16	0.110	0.086
Mean CoP Y axis EC	−20.86	8.45	−17.69	9.96	−2.91	3.26	0.379	0.027

CPE—Conventional Physical Exercise; VR—Vestibular Rehabilitation; SD—Standard Deviation; SE—Standard Error; FIQ—Fibromyalgia Impact Questionnaire; NPRS—Numerical Pain Rating Scale; PCS of SF-12—Physical Component Summary of The 12-Item Short Form Health Survey; MCS of SF-12—Mental Component Summary of The 12-Item Short Form Health Survey; FSS—Fatigue Severity Scale; CSI—Central Sensitization Inventory; TSK—Tampa Scale of Kinesiophobia; PCS—Pain Catastrophizing Scale; DHI—Dizziness Handicap Inventory; ABC-16—Activities-specific Balance Confidence Questionnaire; FES-I—Falls Efficacy Scale International; JAEN Scale—Joint Assessment of Equilibrium and Neuro-motor Scale; HM—Head Movements; SR—Support Reduced; GEO—Gait with Eyes Open; SWEC—Standing and Walking with Eyes Closed; SVV—Subjective Visual Vertical Test; RFT—Rod and Frame Test; RMSX—Root Mean Squared calculated by X axis position values; RMSY—Root Mean Squared calculated by Y axis position values; CoP—Center of Pressure; * *p* < 0.05; ** *p* < 0.01; *** *p* < 0.001.

**Table 4:** Between-group differences by analysis of covariance using baseline scores as covariate. Per-Protocol analysis at 3-month follow-up.

	CPE Group (n = 16)		VR Group (n = 19)		Differences		Effect Size	
	Mean	SD	Mean	SD	Mean	SE	*p*-Value	ETA^2^
FIQ	62.35	18.80	64.58	9.78	0.80	4.29	0.854	0.001
NPRS	6.38	1.96	6.61	1.14	−0.11	0.43	0.794	0.002
PCS of SF-12	38.04	4.66	43.36	5.52	−4.36	1.88	0.027*	0.148
MCS of SF-12	30.32	7.79	24.88	9.58	5.01	3.03	0.108	0.081
FSS	49.06	12.85	46.83	9.79	2.50	3.54	0.485	0.016
CSI	56.63	12.62	60.44	6.40	−0.49	2.34	0.836	0.001
TSK	27.13	6.17	25.67	6.36	0.38	1.42	0.791	0.002
PCS	23.63	12.21	20.44	9.61	2.95	2.04	0.158	0.063
Falls	1.13	1.82	0.39	0.61	0.98	0.44	0.033 *	0.139
DHITotal	54.50	20.66	47.56	15.92	5.18	4.24	0.231	0.046
DHI_Emotional	16.63	9.68	11.67	6.37	2.57	1.60	0.119	0.077
DHI_Functional	19.63	7.84	18.33	6.80	1.19	1.85	0.527	0.013
DHI_Physical	18.25	5.16	17.56	5.55	1.15	1.54	0.458	0.018
ABC-16	57.30	21.84	54.13	17.97	−1.06	5.96	0.860	0.001
FES-I	32.88	10.35	31.83	8.74	−0.02	2.35	0.994	0.000
JAÉN-Total Score	34.44	12.18	25.28	7.92	6.16	3.33	0.074	0.099
JAEN-HM	6.00	4.82	4.67	3.01	1.09	1.29	0.405	0.022
JAEN-SR	21.25	5.88	16.11	5.09	2.26	1.73	0.202	0.052
JAEN-GEO	3.88	2.36	1.94	1.11	1.90	0.57	0.002 **	0.265
JAEN-SWEC	3.31	1.74	2.56	0.86	0.69	0.48	0.163	0.062
SVV	3.48	3.04	2.70	1.17	0.19	0.70	0.783	0.003
RFT	10.10	7.52	6.26	4.40	3.61	1.51	0.024 *	0.164
Sway Area EO	216.50	401.86	112.18	98.12	−37.78	62.75	0.552	0.012
Velocity EO	14.90	3.87	16.42	6.18	−2.65	1.72	0.134	0.076
RMSX EO	0.29	0.06	0.32	0.10	−0.04	0.03	0.220	0.051
RMSY EO	0.31	0.11	0.34	0.16	−0.07	0.04	0.092	0.095
Mean CoP X axis EO	0.23	7.30	−1.06	7.74	1.19	2.45	0.630	0.008
Mean CoP Y axis EO	−19.56	9.82	−14.18	7.15	−3.84	3.18	0.237	0.048
Sway Area EC	254.44	444.79	265.04	341.83	−100.49	101.60	0.331	0.033
Velocity EC	17.94	4.80	18.52	8.61	−1.86	2.45	0.454	0.019
RMSX EC	0.34	0.07	0.37	0.15	−0.05	0.04	0.195	0.057
RMSY EC	0.41	0.14	0.40	0.21	−0.03	0.06	0.637	0.008
Mean CoP X axis EC	0.98	6.56	0.29	6.66	1.08	2.23	0.633	0.008
Mean CoP Y axis EC	−21.78	8.91	−13.55	6.83	−7.88	2.80	0.009 **	0.214

CPE—Conventional Physical Exercise; VR—Vestibular Rehabilitation; SD—Standard Deviation; SE—Standard Error; FIQ—Fibromyalgia Impact Questionnaire; NPRS—Numerical Pain Rating Scale; PCS of SF-12—Physical Component Summary of The 12-Item Short Form Health Survey; MCS of SF-12—Mental Component Summary of The 12-Item Short Form Health Survey; FSS—Fatigue Severity Scale; CSI—Central Sensitization Inventory; TSK—Tampa Scale of Kinesiophobia; PCS—Pain Catastrophizing Scale; DHI—Dizziness Handicap Inventory; ABC-16—Activities-specific Balance Confidence Questionnaire; FES-I—Falls Efficacy Scale International; JAEN Scale—Joint Assessment of Equilibrium and Neuro-motor Scale; HM—Head Movements; SR—Support Reduced; GEO—Gait with Eyes Open; SWEC—Standing and Walking with Eyes Closed; SVV—Subjective Visual Vertical Test; RFT—Rod and Frame Test; RMSX—Root Mean Squared calculated by X axis position values; RMSY—Root Mean Squared calculated by Y axis position values; CoP—Center of Pressure; * *p* < 0.05; ** *p* < 0.01.

## Data Availability

Data used to support the findings of this study are available from the corresponding author upon request.

## Supplementary Materials

The following supporting information Vestibular Rehabilitation (VR) Program can be downloaded at: https://www.mdpi.com/article/10.3390/biomedicines11051297/s1.
