# Supplementary material for: Effectiveness of Vestibular Rehabilitation in Improving Health Status and Balance in Patients with Fibromyalgia Syndrome: A Single-Blind Randomized Controlled Trial
Source: Biomedicines. 2023 Apr 27;11(5):1297. doi: 10.3390/biomedicines11051297 (PMC10216255; doi:10.3390/biomedicines11051297)
Supplement: Supplementary file 1 [file biomedicines-11-01297-s001.zip › biomedicines-2345769-supplementary.pdf]

<table>
<tr><th colspan="5">Vestibular Rehabilitation (VR) Exercises</th></tr>
<tr><th></th><th></th><th>Number</th><th>Exercise</th><th>Exercise Description</th></tr>
<tr><td rowspan="12">LEVEL 1</td><td rowspan="4">Oculomotor exercises (gaze stabilization)</td><td>1</td><td>VORx1 Horizontal</td><td>Arm extended in front and thumb raised. Make head movements on both sides in the horizontal plane, keeping the fixation of the gaze on the thumb for 1 minute. Start sitting and then move to standing. Speed according to tolerance.</td></tr>
<tr><td>2</td><td>VORx1 Vertical</td><td>Arm extended in front and thumb raised. Make head movements up and down, in the vertical plane, keeping the fixation of the gaze on the thumb for 1 minute. Start sitting and then move to standing. Speed according to tolerance.</td></tr>
<tr><td>3</td><td>VORx2 Horizontal</td><td>While still fixing your gaze on your thumb, move your arm extended in the opposite direction in which you move your head on both sides, in the horizontal plane, for 1 minute. Start sitting and then move to standing. Speed according to tolerance.</td></tr>
<tr><td>4</td><td>VORx2 Vertical</td><td>While still fixing your gaze on your thumb, move your arm outstretched in the opposite direction in which you move your head up and down, in the vertical plane, for 1 minute. Start sitting and then move to standing. Speed according to tolerance.</td></tr>
<tr><td rowspan="3">Eye-head movement exercises</td><td>5</td><td>Horizontal tracking with VOR cancellation</td><td>While still fixing your gaze on your thumb, move your arm outstretched in the same direction you move your head, from side to side horizontally, for 30 seconds. Start sitting and then move to standing. Speed according to tolerance.</td></tr>
<tr><td>6</td><td>Vertical tracking with VOR cancellation</td><td>While still fixing your gaze on your thumb, move your outstretched arm in the same direction you move your head, up and down, vertically for 30 seconds. Start sitting and then move to standing. Speed according to tolerance.</td></tr>
<tr><td>7</td><td>Tilt tracking with VOR cancellation</td><td>While still staring at your thumb or an object, tilt your head to one side and the other for 30 seconds. Start sitting and then move to standing. Speed according to tolerance.</td></tr>
<tr><td rowspan="5">Balance and gait exercises</td><td>8</td><td>Feet Together (EO)</td><td>Standing with your feet together, arms along your body and looking horizontally for a minute.</td></tr>
<tr><td>9</td><td>On one leg (EO)</td><td>Standing on one leg, arms along the body and horizontal gaze for 30 seconds. Alternate posture with both feet.</td></tr>
<tr><td>10</td><td>Tandem (EO)</td><td>Feet in line, arms along the body and horizontal gaze for 1 minute.</td></tr>
<tr><td>11</td><td>Ride with head turns</td><td>Walk by turning your head and looking horizontally (100 steps).</td></tr>
<tr><td>12</td><td>Running with horizontal VORx1</td><td>Walking by fixing the gaze on the thumb and head movements on both sides (100 steps).</td></tr>
</table>

		13	**Horizontal tracking gait**	Walking making head turns accompanied by arm movements in the same direction (100 steps).
*Take a 1-minute break between exercises. If there is adequate tolerance of the patient, with low-moderate symptoms of vegetative courtship, the total estimated time for performing Level 1 exercises is 30 minutes.				
**LEVEL 2**	**Oculomotor exercises (gaze stabilization)**	**1**	**VORx1 Horizontal**	Arm extended in front and thumb raised. Make head movements on both sides in the horizontal plane, keeping the gaze fixed on the thumb for 1 min. Decrease base of support until you have your feet together. Increase efficiency to tolerance.
		**2**	**VORx1 Vertical**	Arm extended in front and thumb raised. Make head movements up and down, in the vertical plane, keeping the fixation of the gaze on the thumb for 1 min. Decrease base of support until you have your feet together. Increase efficiency to tolerance.
		**3**	**VORx2 Horizontal**	While still fixing your gaze on your thumb, move your arm extended in the opposite direction to which you move your head on both sides, in the horizontal plane, for 1 min. Decrease base of support until you have your feet together. Increase efficiency to tolerance.
		**4**	**VORx2 Vertical**	While still fixing your gaze on your thumb, move your arm extended in the opposite direction to the one that moves your head up and down, in the vertical plane, for 1 min. Decrease support base until you have your feet together. Increase efficiency to tolerance.
	**Eye-head movement exercises**	**5**	**Horizontal tracking with VOR cancellation**	While still fixing your gaze on your thumb, move your outstretched arm in the same direction you move your head, from side to side horizontally, for 30 seconds. Decrease base of support until you have your feet together. Increase efficiency to tolerance.
		**6**	**Vertical tracking with VOR cancellation**	While still fixing your gaze on your thumb, move your outstretched arm in the same direction as moving your head, up and down vertically, for 30 seconds. Decrease base of support until you have your feet together. Increase speed to tolerance.
		**7**	**Tilt tracking with VOR cancellation**	While still fixing your gaze on your thumb or an object, tilt your head to one side and the other for 30 seconds. Decrease base of support until you have your feet together. Increase speed to tolerance.
		**8**	**Diagonal tracking with VOR cancellation**	While still fixing your gaze on your thumb, move your arm extended in the same direction as moving your head, diagonally for 30 seconds. Decrease base of support until you have your feet together. Increase speed to tolerance. Repeat exercises with opposite diagonal.

		**9**	**Head turning between nearby objects**	Both arms extended in front and thumbs raised, perform fixation on a thumb and then accompany the eye movement with turning of the head in the same direction of the eyes. Alternate eye and head turning movements, in the horizontal plane, for 30 seconds. Decrease base of support until you have your feet together. Increase efficiency to tolerance.
		**10**	**Nodding between nearby objects**	Both arms extended in front, one higher than the other, and thumbs raised, make fixation on a thumb and then accompany eye movement with extension or flexion of the head in the same direction of the eyes. Alternate eye and head movements in the vertical plane, for 30 seconds. Allow support base until you have your feet together. Increase speed to tolerance.
	**Balance and gait exercises**	**11**	**Feet Together (EC)**	Standing with your feet together, arms along your body, and eyes closed, for 1 minute.
		**12**	**On one leg (EC)**	Standing on one leg, arms along the body and eyes closed, for 30 seconds. Alternate posture with both feet.
		**13**	**Tandem (EC)**	Feet in line, arms along the body and eyes closed, for 1 minute.
		**14**	**Nodding gait**	Walk nodding, horizontal gaze (100 steps).
		**15**	**Drive with vertical VORx1**	Walking by fixing the gaze on the thumb and movements of head up and down movements (100 steps).
		**16**	**Vertical tracking gait**	Walking performing head extension flexo movements accompanied by arm movements in the same direction (100 steps).
*Take a 1-minute break between exercises. If there is adequate tolerance of the patient, with low-moderate symptoms of vegetative courtship, the total estimated time for performing Level 2 exercises is 40 minutes. Re recommends reaching a speed during the performance of the exercises of 120bpm/2hz.				
*VOR: vestibulo-ocular reflex; VORx1 and VORx2: Name of specific exercises defined for gaze stabilization and VOR gain; EO: eyes open; EC: eyes closed; Bpm: Beats per minute				
